# Low WSS Induces Intimal Thickening, while Large WSS Variation and Inflammation Induce Medial Thinning, in an Animal Model of Atherosclerosis

**DOI:** 10.1371/journal.pone.0141880

**Published:** 2015-11-17

**Authors:** Antoine Millon, Monica Sigovan, Loic Boussel, Jean-Louis Mathevet, Vanessa Louzier, Christian Paquet, Alain Geloen, Nicolas Provost, Zouher Majd, David Patsouris, Andre Serusclat, Emmanuelle Canet-Soulas

**Affiliations:** 1 Lyon-1 University, CREATIS Laboratory, Lyon, France; 2 Hospices Civils de Lyon, Lyon, France; 3 Lyon-1 University, VetAgro Sup, Lyon, France; 4 Lyon-1 University, CARMEN Laboratory, INSERM U1060, Lyon, France; 5 Genfit Research, Lille, France; University of Washington, UNITED STATES

## Abstract

**Objective:**

Atherosclerotic plaque development in the arterial wall is the result of complex interaction between the wall’s endothelial layer and blood hemodynamics. However, the interaction between hemodynamic parameters and inflammation in plaque evolution is not yet fully understood. The aim of the present study was to investigate the relation between wall shear stress (WSS) and vessel wall inflammation during atherosclerotic plaque development in a minipig model of carotid stenosis.

**Methods:**

A surgical procedure was performed to create left common carotid artery stenosis by placement of a perivascular cuff in minipigs under atherogenic diet. Animals were followed up on 3T MRI, 1 week after surgery and 3, 6, and 8 months after initiation of the diet. Computational fluid dynamics simulation estimated WSS distribution for the first imaging point. Vascular geometries were co-registered for direct comparison of plaque development and features (Gadolinium- and USPIO-Contrast Enhanced MRI, for permeability and inflammation respectively) with the initial WSS. Histological analysis was performed and sections were matched to MR images, based on spatial landmarks.

**Results:**

Vessel wall thickening, permeability and inflammation were observed distally from the stenosis. They were eccentric and facing regions of normal wall thickness. Histological analysis confirmed eccentric plaque formation with lipid infiltration, intimal thickening and medial degradation. High phagocytic activity in the stenosis region was co-localized with high WSS, corresponding to intense medial degradation observed on histology samples.

**Conclusion:**

Lower WSS promotes atherosclerotic plaque development distal to an induced stenosis. Vascular and perivascular inflammation locations were predominant in the high WSS stenosis segment, where medial thinning was the major consequence.

## Introduction

Hemodynamic and metabolic conditions as well as chronic inflammation are key factors in atherosclerotic plaque evolution [1,2].

In mouse models of atherosclerosis, vascular and perivascular macrophage recruitment was observed on non-invasive magnetic resonance imaging (MRI) using ultra-small particles of iron oxide (USPIO) and confirmed by histology [3,4]. It was recently shown that more advanced plaque phenotypes could be obtained by combining atherogenic diet and hemodynamic modifications such as low shear stress [5]. Partial carotid ligation is therefore increasingly recognized as an essential model for studying the relationship between disturbed flow and atherogenesis [6,7]. However, the link between wall shear stress (WSS) and plaque growth and inflammation is still poorly understood [8,9]. Novel mechanically-induced molecular pathways have been identified in the arterial wall in partial-ligation mouse models, but further in-vivo investigations are needed in larger animal models to apply the results to the clinical situation [10]. Both WSS and vessel wall inflammation are involved in plaque development, but have only been evaluated separately on imaging.

Follow-up studies of atherogenesis in murine models using imaging techniques similar to those of clinical protocols are limited by spatial resolution. Consequently, larger animal models, enabling in-vivo imaging using clinical tools, are necessary [11–13]. A previous study in an atherosclerosis pig model has shown a low shear stress and vessel wall thickening in the carotid wall distal to the stenosis [13].

We developed a large animal model of atherosclerosis, combining atherogenic diet and surgically induced local wall shear stress modification, in minipigs. Local lesion evolution was followed sequentially, using a clinical carotid MRI protocol, assessing vessel wall permeability on gadolinium-enhanced MRI and local vessel wall inflammation on USPIO-enhanced MRI. Computational fluid dynamics (CFD) simulations provided detailed analysis of WSS distribution. The study hypothesis was that high WSS and WSS variations could predict the development of local vessel wall inflammation in atherosclerotic plaque.

## Materials and Methods

### Animal model and surgery

Three 1 year-old Göttingen minipigs (Ellegaard, Denmark) were included in the protocol and fed with a high-fat, high-cholesterol diet (750 g to 1 kg/day) for 8 months. After 1 month of this diet, a long stenosis was created in the left common carotid artery by surgical placement of a 1-cm perivascular polytetrafluoroethylene (PTFE) cuff. Pre-medication consisted of intramuscular atropine sulfate (0.025 mg/kg, Aguettant, France, 0.5 mg/mL), azaperone, (2 mg/kg, Stresnilkg, Stresnil, Lure, France), and ketamine (10 mg/kg, Imalg, I 1000, IMerial, Lyon, France). Anesthesia was induced by intravenous injection of 0.03 mL/kg of the following 5mL preparation: 250 mg ketamine (2.5 mL, Imalgmat 1000on Merial, Lyon, France) 20 mg xylazine (2.5 mL, Rompun 2%pun 2% Rompun 2%mg xylazinelazineng 5mLtiletamine with 250 mg of zolazepam (Zoletil 100tilVirbac, Carros, France). Anesthesia was maintained by intravenous injection of 0.015 mL/kg of the same preparation every 30 minutes. The surgical procedure consisted of placement of a perivascular PTFE cuff, 1 cm in length (Figures A and B in S1 File). Heparin was administered during surgery. To prevent thrombosis after surgery, aspirin was given daily at a dose of 150 mg/day for 3 days.

**Fig 1:**
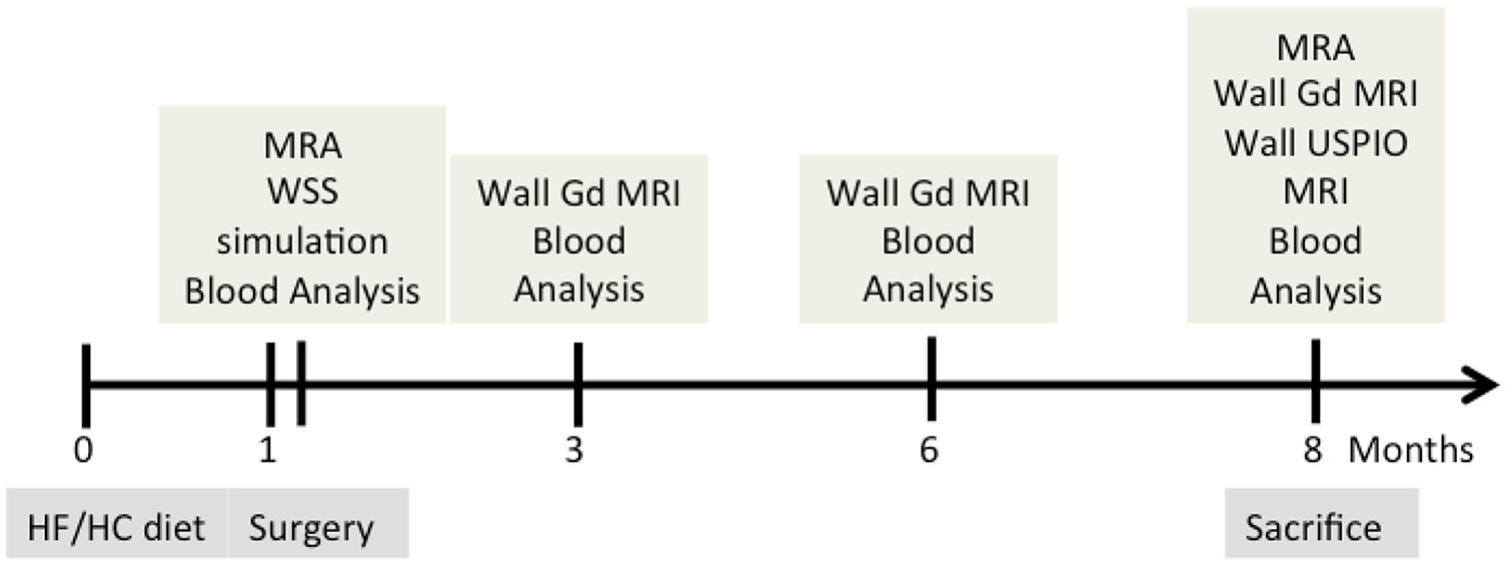
Study flow-chart. **HF/HC diet**: high fat/ high cholesterol atherogenic diet. **Blood analysis**: lipid profile and insulin resistance. **MRA**: MR angiography (stenosis geometry). **Wall Gd MRI**: Gadolinium contrast-enhanced high-resolution MRI (plaque area and vessel wall permeability). **Wall USPIO MRI**: P904 contrast-enhanced high-resolution MRI (vessel wall inflammation).

Blood analysis and MRI were performed 1 week after surgery and 3, 6 and 8 months after initiation of the diet, as shown in the flow chart (Fig 1). After the last MRI exam (showing significant vessel wall thickening), animals were anesthetized and euthanized by injection of T61 (Vetoquinol) for tissue sampling and processing.

The protocol was approved by our institutional review board (approval number #COM056/B).

### Blood and tissue analysis

Blood lipids were analyzed from plasma using standard total cholesterol and HDL cholesterol kits. Lipid profiles were obtained by ultracentrifugation. Insulin sensitivity was evaluated by fasting glycemia and homeostatic model assessment (HOMA IR index). For histology, samples at the level of the stenosis were carefully oriented, using anatomical and ink landmarks (at the distal and frontal carotid wall), for later registration with in-vivo measurements, and fixed in PFA 4% for later processing. Oil Red O staining was performed on 10-μm slices. Tissue macrophages and USPIO uptake were assessed on transmission electron microscopy. Electron microscopy was performed for iron nanoparticle and macrophage imaging. Ultra-thin 30 to 150 nm slices were positioned on a copper grid and examined using a JEOL JEM 1400 microscope equipped with a GATAN Orius SC1000 camera.

### MRI protocol

Imaging was performed on a 3T MRI scanner (Achieva, Philips Medical Systems, Best, and Netherlands); the protocol and sequence parameters are indicated in the Table A in S1 File. Briefly, they consisted of time-of-flight (TOF) and 3D gadolinium-enhanced MR angiography (MRA) acquisitions to obtain luminal geometry, through-plane blood-flow velocity measured on a standard 2D phase-contrast (PC) sequence, and a morphological multi-slice 2D cardiac-gated proton-density (PD) weighted sequence. Gadolinium plaque enhancement was evaluated on high-resolution 2D T1-weighted black-blood sequences acquired before and after MRA. USPIO-enhanced MRI used P904 USPIOs (Guerbet, France; 50 μmol Fe/kg) with high-resolution 2D multi-echo T2* mapping sequences before and 24 hours after USPIO injection, as determined previously in a P904 biodistribution and blood-clearance study in pigs. Kinetics was determined prior to the experiment in three farm pigs by ICP-MS measurement of iron before and for 24 hours after intra-venous injection of 50 μmol Fe/kg (P904, Guerbet, France). Values were corrected for endogenous iron. Pharmacokinetic and biodistribution parameters were measured using standardized curve analysis methods for bi-exponential decay (fast and slow components) (Table B in S1 File).

### CFD simulation

Blood flow analysis used CFD simulation. Animal-specific luminal geometry was obtained by automatic segmentation of the 3D Gd-enhanced MRA data in DicomToolBox. The resulting 3D iso-surface was transferred to Rapidform (INUS Technology), where a polygonal surface was formed. The computational mesh was generated in ANSYS in ANSYS mesh was generated in A IP, Inc.) software. The finite-volume package, FLUENT (ANSYS Inc., Canonsburg, PA) was used to solve the governing Navier-Stokes equations, assuming Newtonian flow with dynamic viscosity of 0.0035 Pa*s and density of 1060 kg/m3. Co-registration of the simulated WSS results to the original 3D MRA was performed for each animal by applying a linear transformation with 9 degrees of freedom using FSL (Analysis Group, FMRIB, Oxford, UK). This method assumes that no changes occurred in the length of the vessels. Pulsatile flow simulation was performed at the first imaging time point (baseline) for each animal. Animal-specific luminal geometry was obtained by automatic segmentation of the 3D Gd-enhanced MRA data. The resulting 3D iso-surface was transferred to Rapidform (INUS Technology), where a polygonal surface was formed. The computational mesh was generated in ANSYS^®^ Workbench TM 2.0 Framework (SAS IP, Inc.) software. The finite-volume package, FLUENT (ANSYS Inc., Canonsburg, PA) was then used, with blood considered as a homogenous Newtonian fluid. Blood velocities were measured on 2D PC MRI by manually drawing regions of interest (ROI) encompassing the vascular lumen, and used as boundary conditions for flow simulation. Iteration steps were repeated up to convergence. Time-averaged WSS was calculated for subsequent analysis.

Vascular geometries obtained at the last imaging time point were co-registered with the baseline geometries with the level of the stenosis as landmark, enabling a direct comparison of local plaque features with baseline WSS.

### Vessel wall analysis

Vessel wall area in both carotid arteries was measured on each co-registered 2D PD-weighted image at 3, 6 and 8 months after left-carotid (LC) surgery. Co-registration was performed with 2D WSS simulation maps with the mid-stenosis point as landmark. Vessel wall area was measured in the LC, and in the right carotid (RC) control, over the entire slice stack (eight 2-mm PD slices every 5 mm). To study vessel wall remodeling over time, the stack was divided into distal and proximal segments, with the latter encompassing the stenosis. Slices exceeding 2-mm thickness were considered atherosclerosis-positive. In eccentric plaque, the thickened region, considered atherosclerosis-positive, was compared to the facing atherosclerosis-negative region and to the control RC wall. For local circumferential WSS analysis, atherosclerosis-positive walls with eccentric plaque were similarly separated into two regions of interest (ROI) delineated on the PD image as “plaque-positive” and “plaque-negative”. Gadolinium contrast enhancement (CE) was used to improve plaque visualization and assess vessel wall permeability. Gadolinium signal intensity (SI) in the plaque-positive ROI was measured longitudinally at 3, 6 and 8 months on pre- and post-contrast T1-weighted images. Vessel wall gadolinium CE ratio was assessed by comparing post- and pre-contrast SI, normalized to the SI in the adjacent sternocleidomastoid muscle with the following equation: T1 CE ratio = (SI plaque post×SI muscle pre)/(SI plaque pre×SI muscle post). Again, co-registration with 2D WSS measurements was performed.

### Vessel wall inflammation measurement

When significant thickening (>2mm thickness) was observed on MRI, vascular macrophage activity was assessed on USPIO-enhanced MRI. Post-USPIO change in SI was first visually assessed on 3D MRA as area of signal-loss along the arterial lumen, and then analyzed by comparing T2* measurements (in msec) from T2* maps acquired before and 24 hours after USPIO injection. T2* maps were analyzed at the same levels and with the same ROIs as for gadolinium enhancement.

### Statistics

The Pro JMP 11.0 statistical package (SAS Institute Inc., Cary, NC, USA) was used for all analyses. Two-group comparison used a Mann–Whitney nonparametric test for continuous variables. Multiple-group comparison used 1-way ANOVA followed by non-parametric Mann–Whitney post-hoc test. Data are shown as means ± standard errors of the mean (sem). p<0.05 was considered statistically significant.

## Results

**Fig 2:**
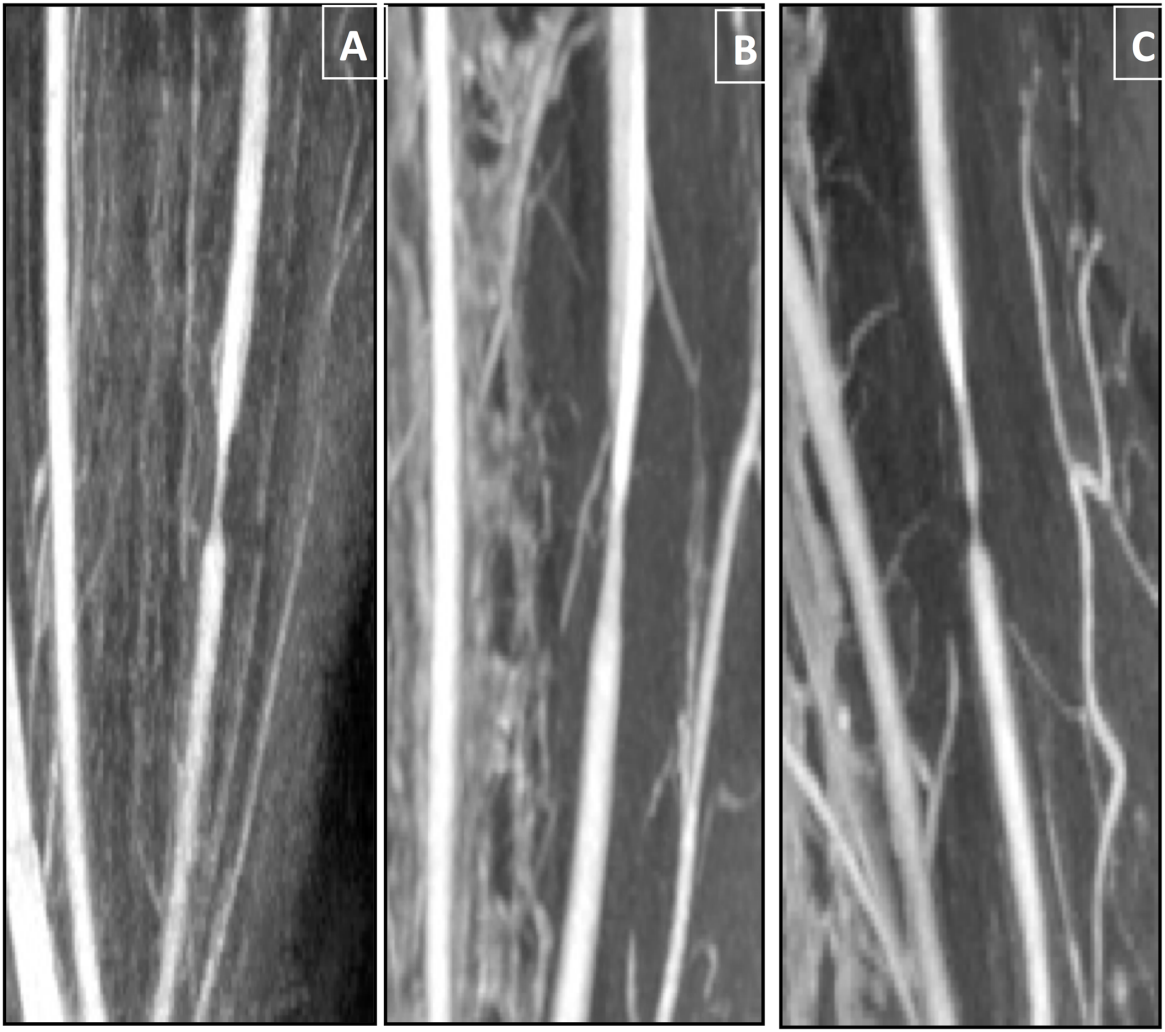
A-C: Baseline geometry of the left carotid artery stenosis on MRI angiography for the 3 animals: respectively 83%, 63% and 74% stenosis for animals #1, #2 and #3 (from left to right).

All three animals showed increased body-weight over the 8 months' follow-up and developed severe hypercholesterolemia, demonstrated mainly by increased low-density lipoprotein (LDL) concentration, from 105±68 mg/dL at baseline to 296±108 mg/dL at 8 months (Figures A and B in S2 File). After 8 months' diet, they presented high levels of free fatty acids (0.8±0.3 mmol/L), but no marked hypertriglyceridemia (99±25 mg/dL) and no insulin resistance (glycemia 79.4±5 mg/dL and HOMA IR index 1.3±0.3). All MRI protocols were successfully completed for each time point, and all animals showed significant stenosis on MRA (83%, 63% and 74% stenosis for animals #1, #2 and #3 respectively) (Fig 2 and Figures C and D in S2 File).

### Atherosclerosis development and WSS

**Fig 3:**
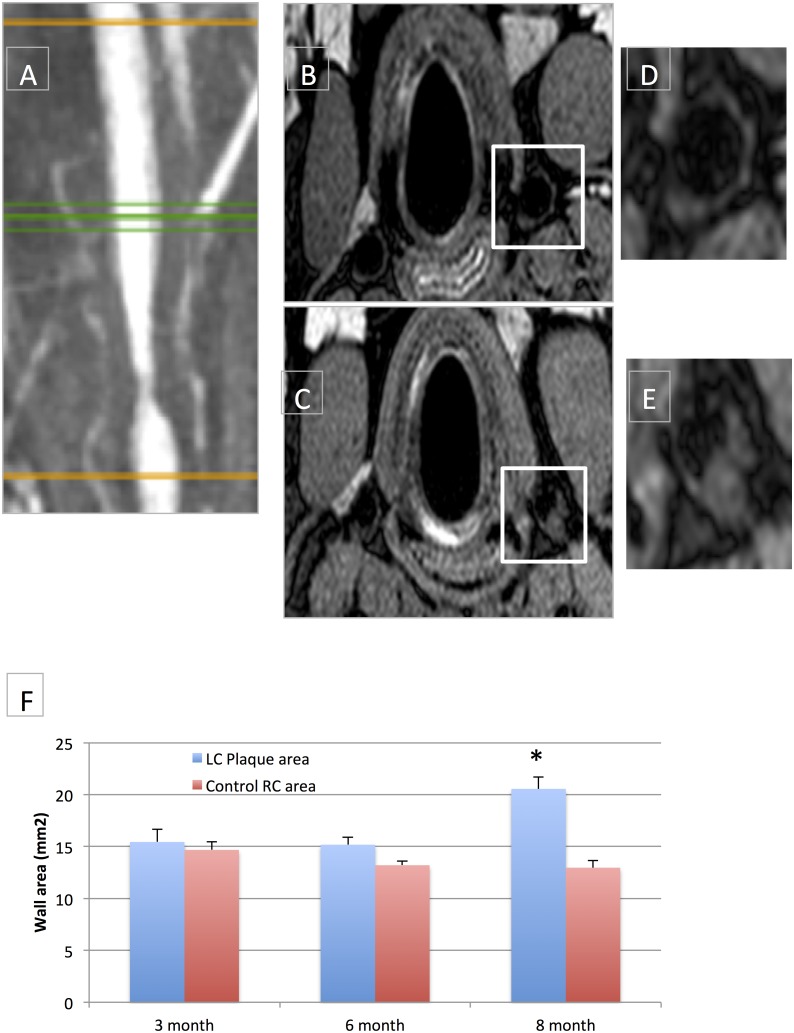
A-F: Final MRI stenosis geometry and corresponding measurement of vessel wall thickening compared to the control right carotid artery. **A**. 8-month MRA of animal#1 and location of proton density slices for distal vessel wall thickness measurement (green line). **B-E**. Proton density transverse images of animal #1, showing the evolution of left and right carotid (LC and RC) distal wall thickness, from 3 (B) to 8 month (C). **D-E**. Larger view of the LC wall at 3 (D) and 8 month (E). **F**. Longitudinal vessel wall measurement in the LC and control RC for the three animals, showing significant (*) difference at 8 month.

**Fig 4:**
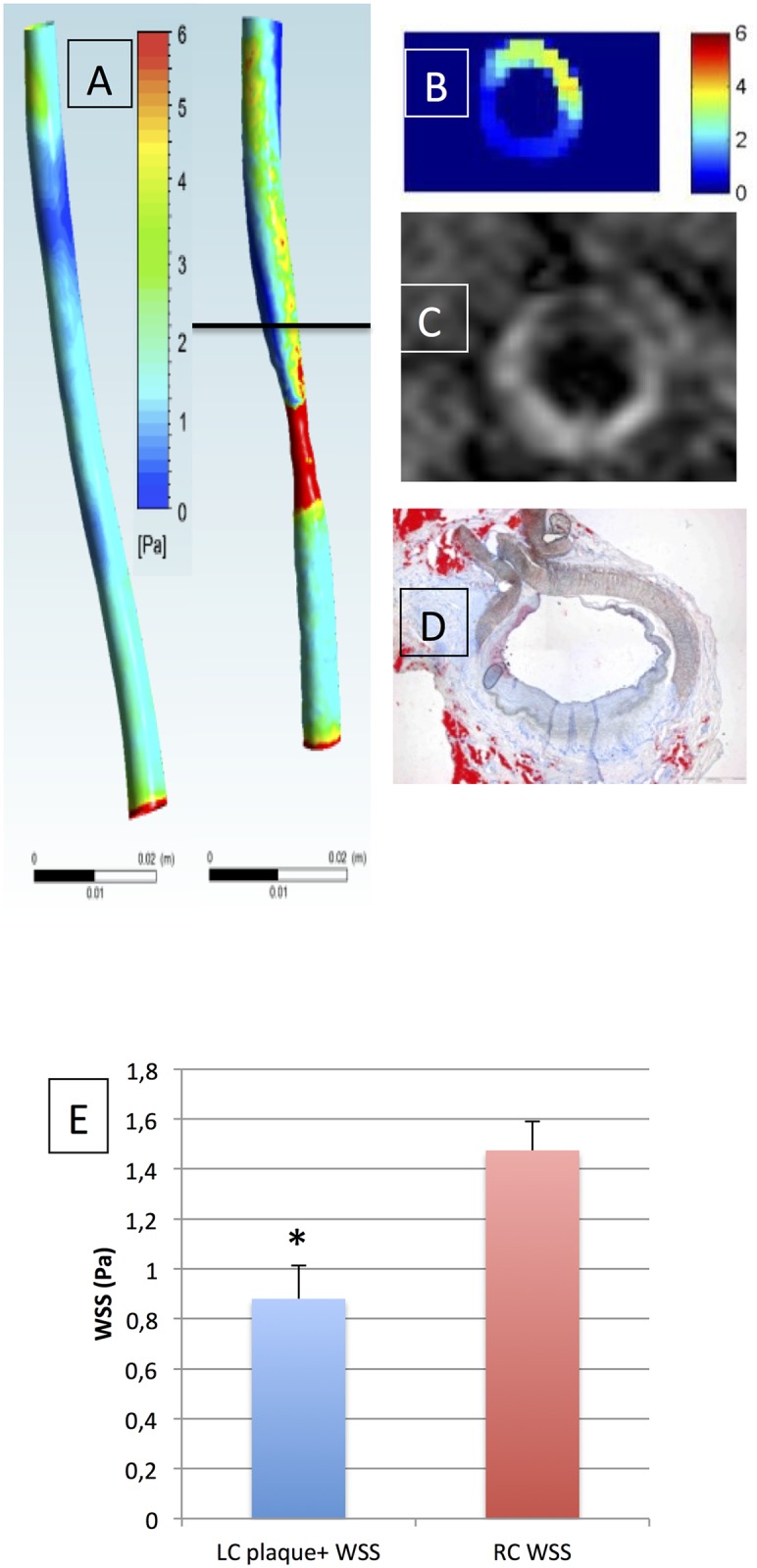
A-E: Lower wall shear stress and post-gadolinium enhancement in the plaque-positive distal portion of the left carotid artery. **A-C**. 3D WSS of animal #2 obtained by CFD in the right (RC) and left (LC) carotids (A); and location-matched (black line in A) WSS LC cross-section, showing transverse distribution of lower WSS areas (B). **C-D** Co-localized positive contrast on the post-Gd T1-weighted sequence (at 8 months) (C), and intimal thickening and lipid infiltration in the corresponding Oil Red O slice (D). **E**. Mean WSS values (Pa) corresponding to spatially matched regions of distal LC wall (plaque-positive ROIs) and control RC wall, showing lower values in the distal LC slices compared to control RC (* significant difference).

**Fig 5:**
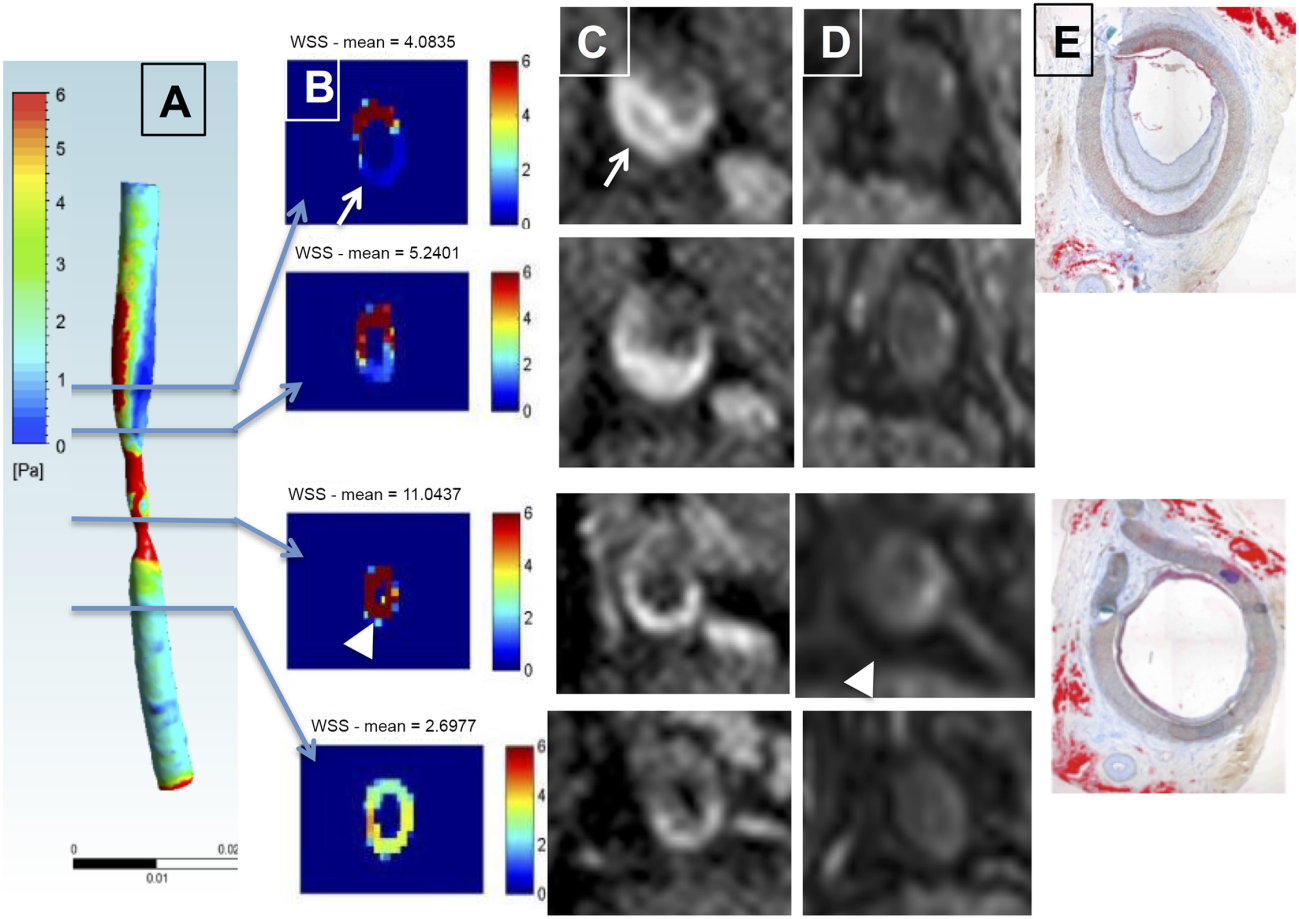
A-F: WSS, gadolinium enhancement and post-USPIO phagocytic activity, showing parallel heterogeneous patterns on four transverse slices. **A**. 3D WSS representation for animal #3 LC. **B**. Transverse WSS maps in the stenosis and post-stenosis segments with respectively high (arrow head) and low WSS (arrow) areas. **C**. Corresponding post-gadolinium T1 slices, showing larger vessel wall enhancement regions in the post-stenosis segments at 8 months (arrow). **D**. Corresponding post-USPIO T2* maps showing vascular and perivascular inflammation in the stenosis (arrow head) and post-stenosis segments. **E-F**. Two corresponding Oil Red O slices, showing eccentric intimal thickening and medial thinning on the opposite side (E, end-stenosis, distal) and lipid infiltration with medial thinning (F, stenosis level).

Eccentric LC wall thickening distal to the stenosis was observed at 8 months on PD images (20.6±1.1 mm^2^ wall area) compared to the RC control (12.9±0.7 mm^2^ wall area, p<0.002) (Fig 3). “Plaque positive” LC distal wall thickening corresponded to regions of low WSS (0.88±0.13 N/m^2^) compared to the normal wall of the RC control (1.47±0.11 N/m^2^, p<0.01). The “plaque-negative” LC ROI presented higher WSS values (4.07±1.21 N/m^2^, p<0.01). However, the highest WSS values were found at the level of the stenosis, with a mean 23.87±6.28 N/m^2^ for the three animals, with large longitudinal and circumferential variation, as seen on the coronal and transverse views of WSS distribution (Figs 4 and 5). Gadolinium enhancement demonstrated increased vessel wall permeability in the plaque-positive areas of the distal LC wall, with high enhancement ratios (2.21±0.16) compared to plaque-negative areas (1.88±0.4).

### Vessel wall inflammation and WSS

**Fig 6:**
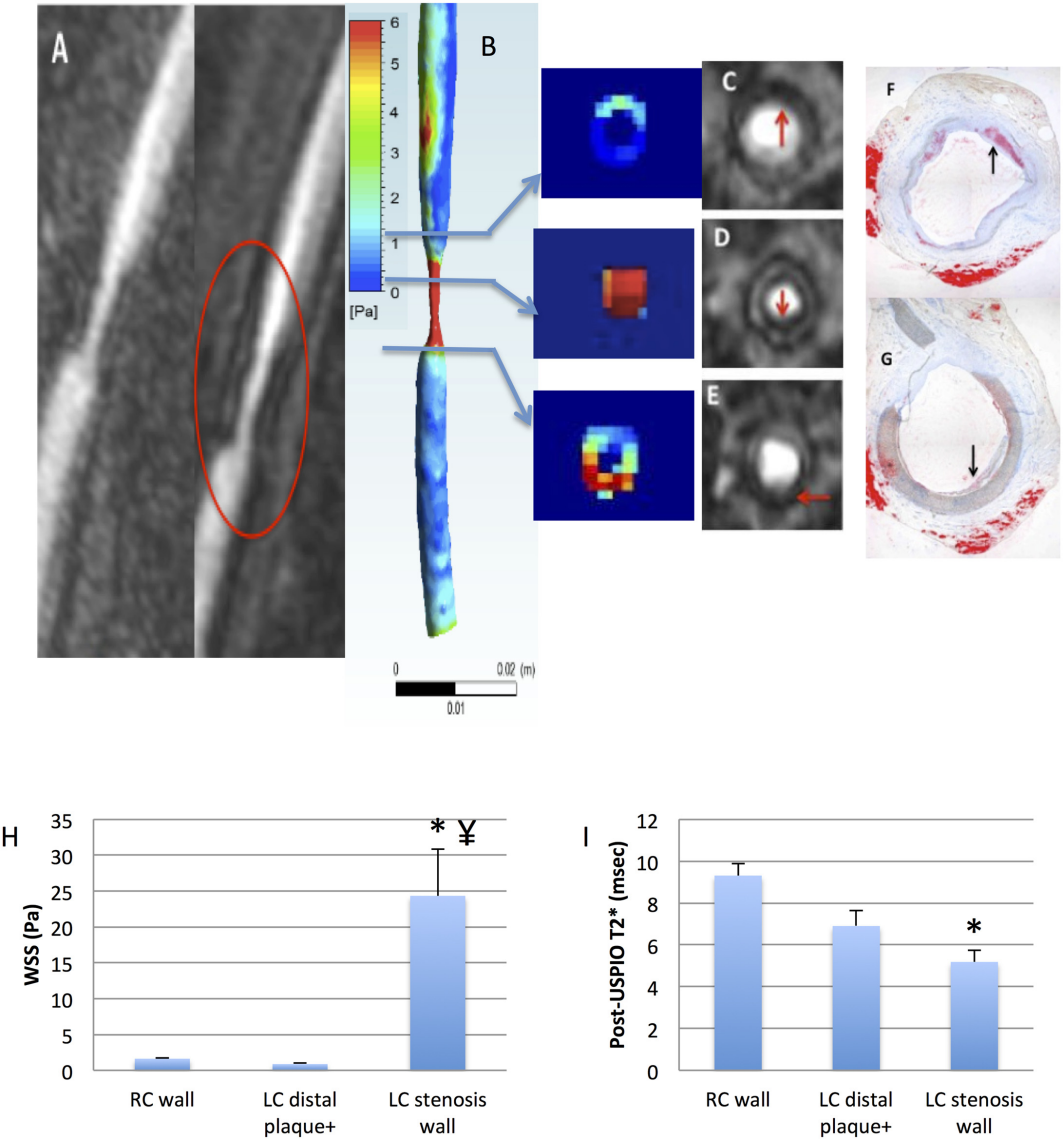
A-I: WSS and phagocytic activity distribution in stenosis and post-stenosis segments. **A**. Pre and post-USPIO CE MRA of the left carotid (animal#1 at 8 month), showing low-intensity areas in the stenosis. **B**. Corresponding 3D WSS and transverse maps, showing strong longitudinal and circumferential WSS heterogeneity. **C-E**. Reformatted transverse MRA slices, showing locations of low intensity areas (red arrows). **F-G**. Corresponding Oil Red O stained histological slices, with intimal thickening (F) and medial thinning (G), respectively (black arrows). **H**. Mean WSS values (N/m^2^) corresponding to spatially matched regions of distal LC wall (plaque-positive ROIs), control RC wall, and LC wall in the stenosis (*, significantly different from control RC wall, ¥ significantly different from distal plaque+ LC wall). **I**. T2* values (ms) corresponding to spatially matched regions of distal LC wall (plaque-positive ROIs), control RC wall, and LC wall in the stenosis (* significantly different from control RC).

At the last imaging point, the T2* decrease observed on post-USPIO images indicated pronounced phagocytic activity, and thus vessel wall inflammation, along the LC wall compared to the RC control (6.1±0.3 msec vs. 9.3±0.6 msec, p<0.02; and 15.5±0.2 msec in the control muscle, p<0.0001). The T2* effect was stronger in regions with large WSS variation (Figs 5 and 6). These regions corresponded to pronounced medial degradation on histology (Fig 6). For one animal, USPIO-enhanced MRI was performed at 3, 6 and 8 month. The T2* effect was observed in the stenosis segment on post-USPIO MRI at each time point (Figure E in S2 File).

### Histology

All animals developed plaques characterized by eccentric intimal thickening in the segment distal to the stenosis. Inside the stenosis, extreme medial thinning was observed, corresponding to regions of high WSS and high phagocytic activity on MRI. Postmortem examination revealed additional atherosclerotic plaque locations (iliac, coronary and aorta). Macrophages laden with lipids and iron oxide particles were observed on electron microscopy in both the vascular lesions and perivascular fat. Perivascular fat analysis revealed a shift in adipocyte size at the stenosis compared to the RC control: i.e., another indicator of chronic inflammatory activity (Figure F in S2 File).

## Discussion

In this study, two atherosclerosis processes, intimal thickening and medial thinning, were both linked to abnormal hemodynamics and inflammation at, respectively, two distinct locations. As previously demonstrated in the minipig model, we confirmed that intimal plaque developed distally to the stenosis, induced by low WSS and correlating with vessel wall permeability [13]. The present study demonstrated for the first time that vascular inflammation within the stenosis, persisting at 8 months, leads to extreme medial thinning. This high phagocytic activity was collocated with very large WSS variations.

Although the entire vasculature is exposed to the atherogenic effects of systemic risk factors, atherosclerotic lesions develop in specific regions of the arterial tree: near bifurcations, bends and branch ostia. Hence, local hemodynamic factors, and in particular low endothelial shear stress, play a fundamental role in the location of atherosclerotic plaques [14,15]. CFD study of hemodynamic factors such as WSS is now considered reliable. Generating hemodynamic disturbances in animal models exposed to systemic factors (hypercholesteremia, diabetes, hypertension) accelerates the process of atherosclerosis. Different techniques for generating the disturbance have been described [11,16–18]; inserting a perivascular collar has the advantage of maintaining the structural integrity of the endothelium while inducing rapid, site-controlled lesion formation [19,20]. Cheng et al., in an animal model of atherosclerosis using a perivascular shear stress modifier in mouse aorta and rabbit carotid arteries [5,21], observed decreased shear stress upstream of the cast, increased shear stress inside the cast and low-oscillatory shear stress downstream of the cast. Atherosclerotic lesions developed in regions with low and oscillatory shear stress whereas regions of increased shear stress were protected. Lesions in regions with low shear stress (upstream of the cast) were more extensive and more vulnerable than those in regions with oscillatory shear stress (downstream of the cast).

More recently, Thim et al. placed a silicon collar on the carotid of hypercholesterolemic minipigs 4 weeks after initiating atherogenic diet [7]. Atherosclerotic lesions were observed in the post-stenotic segment, where low and oscillatory shear stresses were present simultaneously. In patent carotid arteries, no lesions were observed in the pre-stenotic segment, where only low shear stress was observed.

The present results are in agreement with those of Thim et al. MRI at 3, 6 and 8 months found atherosclerotic lesions in the post-stenotic segment, spatially correlating with low baseline WSS. We did not observe any lesions in the pre-stenotic segment. Intima thickening started at the end of the cuff segment, where there was also strong degradation of the tunica media. This level was characterized by large variations in both longitudinal and circumferential WSS, indicating that low WSS was not the only hemodynamic disturbance related to arterial lesions. This is in agreement with a recent review by Peiffer et al., where the authors stated that the evidence for the low/oscillatory shear stress theory is less robust than commonly assumed [22]. The consequence of high WSS on vessel wall biology, as well as the relative importance of oscillatory shear stress in these high WSS regions, is still a matter of debate [22, 23].

Gadolinium enhancement in the vessel wall may be related to increased permeability or to increased perfusion through leaky neovessels [24,25]. The present study found gadolinium enhancement within the plaque compared to the adjacent vessel wall. Histology, however, did not reveal any neovasculature. The enhancement therefore seems to be related to increased permeability of the endothelial layer under low wall shear stress. This relationship between low shear stress and increased endothelial permeability was previously demonstrated in a porcine model [26,27]. The present results support these initial studies, and demonstrate the feasibility of observing this low WSS effect on in-vivo imaging.

Signal change after P904 USPIO injection is a known indicator of an inflammatory process with increased phagocytic activity [28,29]. The present results showed a strong T2* decrease after P904 injection at three time points, indicating chronic inflammation along the left carotid in contrast to the right carotid control. The strongest T2* effect was observed at the stenosis, corresponding to high WSS regions and the strong medial degradation observed on histology. The inflammation and media thinning observed in the stenosis region may be related to an atherosclerotic process. This hypothesis is supported by the study by Moreno et al., who demonstrated that preatheroma (class III) lesions showed increased medial fibrosis and decreased medial thickness [30]. Furthermore, disturbed WSS induces transcriptional changes, not only in endothelial cells [31] but also in vascular smooth muscle cells [32], responsible for arterial wall inflammation and inducing medial degradation [33], as seen in the formation of aneurysms. The relationship between increased WSS and plaque erosion atherosclerotic plaque rich in smooth muscle cells was recently demonstrated in a rabbit model [23]. The only study combining biomechanical stress analysis and USPIO-enhanced MRI assessment of vessel wall inflammation showed that increased maximal stress corresponded to increased signal loss [34].

Another hypothesis to explain such lesions is tunica media necrosis induced by the perivascular cuff, which may impair vessel wall vascularization from the adventitia and perivascular tissue, leading to necrosis and inflammation.

The small number of animals is a potential limitation of the present study. However, all three animals had a similar response to diet-induced hypercholesterolemia and successful stenosis, inducing significant changes in WSS and the development of plaques. Moreover, as there were many imaging time points, each animal served as its own control thanks to their control right carotid artery (see Supporting Information), respecting animal experimentation guidelines to reduce the number of animals, especially in large-animal models.

Another limitation was that averaging CFD over the cardiac cycle did not allow the oscillatory component of WSS to be assessed.

Future biological experiments may provide deeper understanding of the mechanisms underlying vascular and perivascular inflammation in stenosis.

In conclusion, the present results illustrate the interest of longitudinal MRI follow-up of the atherosclerotic process, using a combination of morphological analysis, computer modeling and functional imaging biomarkers (permeability and inflammation).

## Supporting Information

S1 File Supplemental Methods. Figures A-B display surgical placement of the perivascular collar on the common left carotid artery. Table A summarizes the MRI sequence parameters and Table B the P904 pharmacokinetic parameters in pigs at 50 **μ**mol Fe/kg.
(DOCX)

S2 File Supplemental results. Figures A and B display Total cholesterol, LDL cholesterol and lipoprotein profile evolution. Figures C and D display Left carotid stenosis geometry over time measured from 3D MRA, Control right carotid wall area over time and corresponding WSS. Figure E displays Presence of vascular and perivascular inflammation at the stenosis over time, followed by post-USPIO CE MRA at 3, 6 and 8 months (animal #3). Figures F (A-G) display additional histological results.
(DOCX)
