# Supplementary material for: Low WSS Induces Intimal Thickening, while Large WSS Variation and Inflammation Induce Medial Thinning, in an Animal Model of Atherosclerosis
Source: PLoS One. 2015 Nov 17;10(11):e0141880. doi: 10.1371/journal.pone.0141880 (PMC4648591; doi:10.1371/journal.pone.0141880)
Supplement: S1 File — Figures A-B display surgical placement of the perivascular collar on the common left carotid artery. Table A summarizes the MRI sequence parameters and Table B the P904 pharmacokinetic parameters in pigs at 50 μmol Fe/kg. (DOCX) [file pone.0141880.s001.docx]

**Supporting Information File 1: Supplemental Method**

**Figures A-B**: Surgical placement of the perivascular collar on the common left carotid artery. A: dissection around the artery. B: Position of the cuff around the artery maintained by two sutures.

**A B**


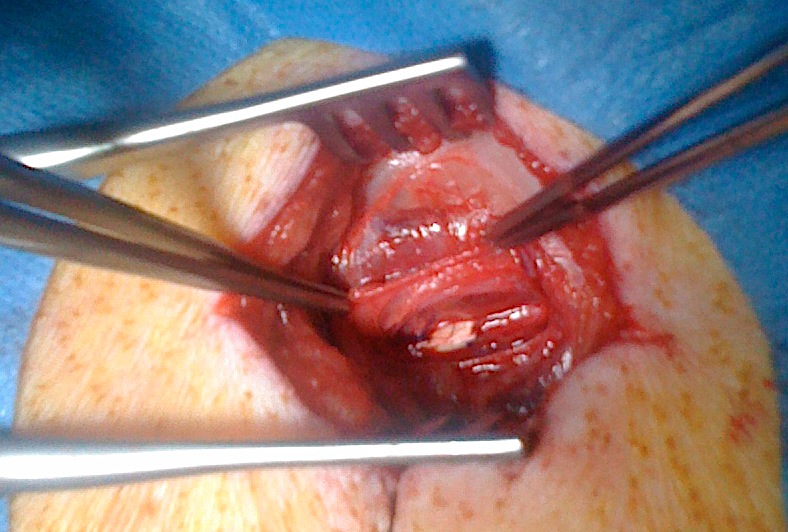

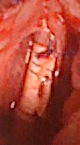


**Table 1: MRI sequence parameters**

|  | TR (ms) | TE (ms) | Flip angle | Bandwidth (Hz/pixel) | ST (mm) | Matrix size |
| --- | --- | --- | --- | --- | --- | --- |
| DP-trig | 2000 | 33.4 | 90 | 381 | 2 | 560*560 |
| T1-BB-trig Sense Fat Saturation (6 averages) | 1000 | 23 (Echo train length =9) | 90 | 276 | 2 | 560*560 |
| TrigIP-T2*-map Sense (6 averages) | 18.3 | 2.2-16 (5 echoes) (Echo train length =65) | 30 | 788 | 2 | 320*320 |
| TSA | 5.2 | 2 | 27 | 560 | 0.49 | 768*768 |
| sQflow | 8.4 | 5.6 | 15 | 528 | 6 | 448*448 |

**Table 2: P904 pharmacokinetic parameters in pigs at 50 µmol Fe/kg**

|  | Fast T_1/2_ (min) | Slow T_1/2_ (h) | Vd (µmoLFe.kg^-1^) | Clearance (µmoLFe.kg^-1^.h^-1^) |
| --- | --- | --- | --- | --- |
| 50µmolFe/kg (injected volume 2.2±0.1 mL) | 124±40 | 34±7 | 0.1±0.005 | 0.002±0.0005 |
