# Supplementary material for: Low WSS Induces Intimal Thickening, while Large WSS Variation and Inflammation Induce Medial Thinning, in an Animal Model of Atherosclerosis
Source: PLoS One. 2015 Nov 17;10(11):e0141880. doi: 10.1371/journal.pone.0141880 (PMC4648591; doi:10.1371/journal.pone.0141880)

**Supporting Information File S2: Supplemental Results**

**Figures A and B: Total cholesterol, LDL cholesterol (A) and lipoprotein profile evolution (B)**

1. Total Cholesterol and LDL cholesterol (measured using the Friedewald formula) over time for the 3 animals under high fat diet, compared to a control lean Göttingen minipig.

1. Evolution of lipoprotein profile over the 8 months of diet from the first MRI time point (blue trace) to the last (purple trace), showing the huge increase in LDL peak for all three animals.


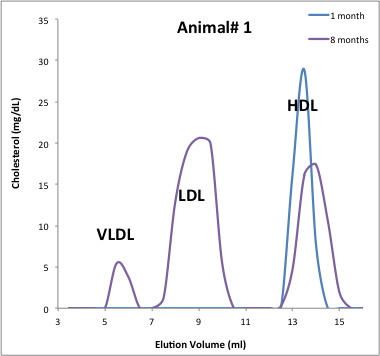

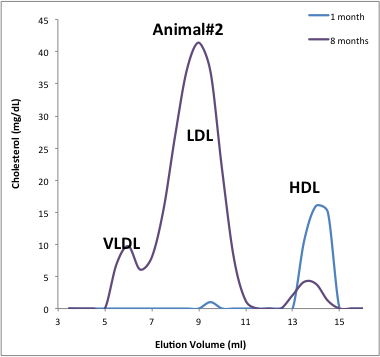

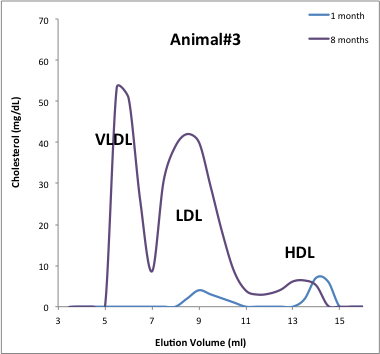


**Figures C and D: Left carotid (LC) stenosis geometry over time measured from 3D MRA (C), Control right carotid wall area over time and corresponding WSS (D)**

1. The stenosis geometry (length and severity) over time is measured from 3D MRA, the stenosis severity degree is defined as 100*(1-(stenosis diameter/proximal diameter)). The mean length of the stenosis is 0.79±0.04 cm and the mean stenosis degree 68.5±2.5 %


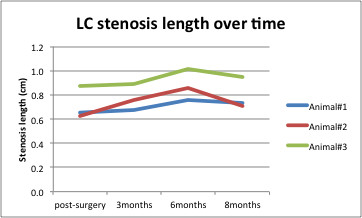

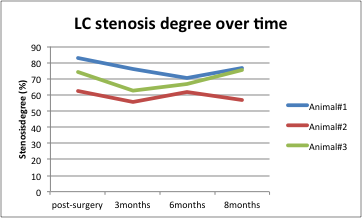


1. Quartile distributions of right carotid (RC) wall area over time (n=7 to 8 slices per animal per time point) and RC wall shear stress (WSS) in the three animals (n=8 measurements, corresponding to slice locations, per animal)


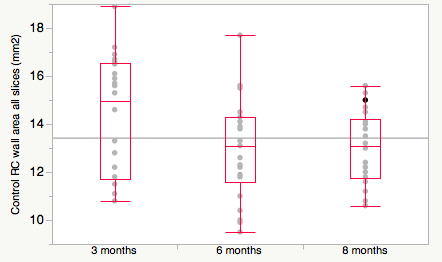


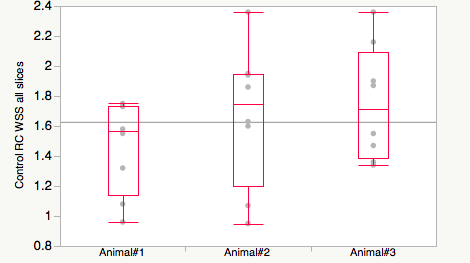


**Figure E: Presence of vascular and perivascular inflammation (arrows) at the stenosis over time, followed by post-USPIO CE MRA at 3, 6 and 8 months (animal #3)**

Both left control right carotids are shown on the left, and the magnified stenosis view is shown on the right. LC T2* values on corresponding T2* maps found a decrease from 27.8 msec to 4.6 msec, 24 hours after USPIO injection.

***
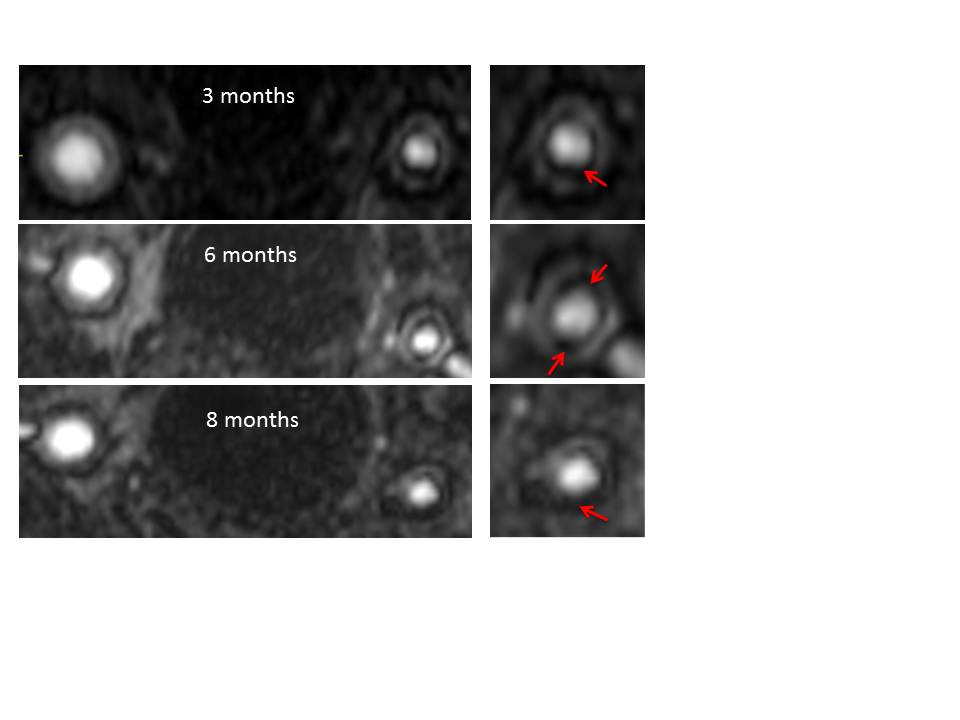
***

**Figures F: Histological analysis**

**A-B**. Histological confirmation of multi-site plaque location in carotid (A) and coronary (B) arteries, showing lipid infiltration, medial thinning and intimal thickening in both vessels (animal #3: carotid and coronary ORO slices).

**C-D**. Multi-site inflammation: TEM showing macrophage-laden iron oxide particles in the liver (C, with enlarged view showing iron particles) and iron oxide particles in the iliac artery (D).

**E-F**. Circulating lipid-laden macrophages at the level of the carotid plaque (E) and perivascular fat remodeling, with large adipocytes and lipid-laden perivascular macrophages at the level of the iliac plaque (F).

**G.** Perivascular fat remodeling is confirmed by peri-stenosis changes in adipocyte size, as shown by the rightward shift in cell size distribution.


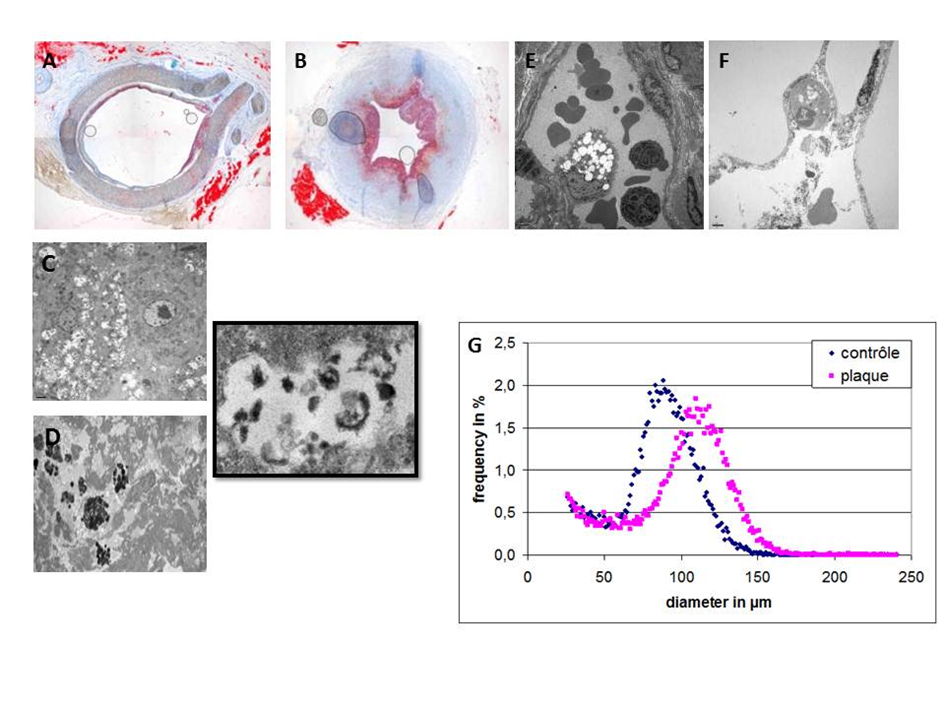

Supplement: S2 File — Figures A and B display Total cholesterol, LDL cholesterol and lipoprotein profile evolution. Figures C and D display Left carotid stenosis geometry over time measured from 3D MRA, Control right carotid wall area over time and corresponding WSS. Figure E displays Presence of vascular and perivascular inflammation at the stenosis over time, followed by post-USPIO CE MRA at 3, 6 and 8 months (animal #3). Figures F (A-G) display additional histological results. (DOCX) [file pone.0141880.s002.docx]
